# A novel mutation in *TTC19* associated with isolated complex III deficiency, cerebellar hypoplasia, and bilateral basal ganglia lesions

**DOI:** 10.3389/fgene.2014.00397

**Published:** 2014-11-14

**Authors:** Laura Melchionda, Nadirah S. Damseh, Bassam Y. Abu Libdeh, Alessia Nasca, Orly Elpeleg, Alice Zanolini, Daniele Ghezzi

**Affiliations:** ^1^Unit of Molecular Neurogenetics, Foundation IRCCS Istituto Neurologico Carlo BestaMilan, Italy; ^2^Genetic Unit, Al-Makassed Islamic Charitable HospitalJerusalem, Israel; ^3^Monique and Jacques Roboh Department of Genetic Research, Hadassah – Hebrew University Medical CenterJerusalem, Israel

**Keywords:** *TTC19*, complex III deficiency, novel mutation, mitochondrial diseases, bilateral basal ganglia lesions, encephalomyopathy

## Abstract

Isolated complex III (cIII) deficiency is a rare biochemical finding in mitochondrial disorders, mainly associated with mutations in mitochondrial DNA *MTCYB* gene, encoding cytochrome b, or in assembly factor genes (*BCS1L*, *TTC19, UQCC2,* and *LYRM7*), whereas mutations in nuclear genes encoding cIII structural subunits are extremely infrequent. We report here a patient, a 9 year old female born from first cousin related parents, with normal development till 18 months when she showed unsteady gait with frequent falling down, cognitive, and speech worsening. Her course deteriorated progressively. Brain MRI showed cerebellar vermis hypoplasia and bilateral lentiform nucleus high signal lesions. Now she is bed ridden with tetraparesis and severely impaired cognitive and language functions. Biochemical analysis revealed isolated cIII deficiency in muscle, and impaired respiration in fibroblasts. We identified a novel homozygous rearrangement in *TTC19* (c.213_229dup), resulting in frameshift with creation of a premature termination codon (p.Gln77Argfs*30). Western blot analysis demonstrated the absence of *TTC19* protein in patient’s fibroblasts, while Blue-Native Gel Electrophoresis analysis revealed the presence of cIII-specific assembly intermediates. Mutations in *TTC19* have been rarely associated with mitochondrial disease to date, being described in about ten patients with heterogeneous clinical presentations, ranging from early onset encephalomyopathy to adult forms with cerebellar ataxia. Contrariwise, the biochemical defect was a common hallmark in *TTC19* mutant patients, confirming the importance of *TTC19* in cIII assembly/stability. Therefore, we suggest extending the *TTC19* mutational screening to all patients with cIII deficiency, independently from their phenotypes.

## INTRODUCTION

Mitochondrial respiratory chain consists of five enzymatic multi-heteromeric complexes embedded in the inner membrane of mitochondria. Complex III (cIII) is responsible for the electron transfer from reduced Coenzyme Q to cytochrome *c*. It consists of 11 subunits, one of which (cytochrome *b,* cyt *b*) is encoded by the mitochondrial DNA (mtDNA), whereas the others are encoded by nuclear genes, synthesized in the cytosol and imported into mitochondria (Iwata and Nakai, 1998). cIII assembly is a step-wise process, requiring the action of dedicated assembly factors (Ghezzi and Zeviani, 2012).

About half of mitochondrial disorders with cIII deficiency (MIM124000) remains without a molecular diagnosis and this is principally due to the incomplete understanding of cIII assembly and to a wide phenotypic heterogeneity of patients. Isolated cIII deficiency is mainly associated with mutations in mtDNA *MTCYB* gene (MIM516020), encoding cyt *b*, or in assembly factor genes (*BCS1L–*MIM603647, *TTC19–*MIM613814*, UQCC2–*MIM614461, and *LYRM7–*MIM615831), whereas mutations in nuclear genes coding cIII structural subunits are extremely infrequent. Most of the *MTCYB* mutations are sporadic and cause a mitochondrial myopathy. cIII disorders due to nuclear mutations have usually an autosomal recessive inheritance pattern with onset at birth. The peculiar clinical features are lactic acidosis, hypotonia, failure to thrive, delayed psychomotor development, encephalopathy (Ghezzi and Zeviani, 2012; Invernizzi et al., 2013); in some patients visceral involvement as hepatopathy and tubulopathy has been reported (de Lonlay et al., 2001; De Meirleir et al., 2003; Tucker et al., 2013).

*TTC19* is a mitochondrial protein, embedded in the inner mitochondrial membrane, which has been proposed to have a role in an early step of cIII assembly. Mutations in *TTC19* were described for the first time in three Italian individuals from two unrelated families with young-onset characterized by slowly progressive encephalopathy and isolated cIII deficiency, and in a fourth Italian individual with an adult-onset characterized by subacute, rapidly progressive neurological failure and isolated cIII deficiency (Ghezzi et al., 2011). After this paper few other patients have been reported with *TTC19* mutations, all presenting isolated cIII deficiency, but various clinical-radiological phenotypes: severe olivo–ponto–cerebellar atrophy and progressive psychosis in Portuguese siblings from a consanguineous family (Nogueira et al., 2013), Leigh syndrome in a Hispanic 4 year-old boy (Atwal, 2013), or adult-onset spinocerebellar ataxia in a Japanese woman (Morino et al., 2014).

Here, we report a novel deleterious mutation in *TTC19* in a patient (Pt) with cIII deficiency, tetraparesis, cerebellar, and basal ganglia abnormalities.

## MATERIALS AND METHODS

### CASE REPORT

We describe a 9 year-old female child from Palestinian related parents (first cousins) of Muslim origin. Pregnancy and delivery were unremarkable; the baby weighted 3 kg at birth. She was referred to have had normal development until 18th month of age, when parents started noticing unsteady gait with frequent falling. These symptoms were not cared of until 7 years of age, when learning difficulties became evident: she showed attention and memory deficit with difficulties manipulating objects and dysgraphia. Behavioral alterations were also noted, with abnormal sleep pattern characterized by night terrors and purposeless movements.

At first evaluation (at 7 years of age) she weighted 18 kg (5th centile), her height was 116 cm (25th centile), and her head circumference was normal (51.5 cm). Physical examination revealed neither facial dysmorphisms nor organomegaly. Neurological examination showed normal eye movements, cock-wheel rigidity of upper limbs with normal tone of legs, unsteady wide-based gait. Deep tendon reflexes were normal. Her blood and urine work including lactate, ammonia, CPK, hepatic assessment, urine organic acids, and plasma amino acid chromatography did not show any alteration.

**FIGURE 1:**
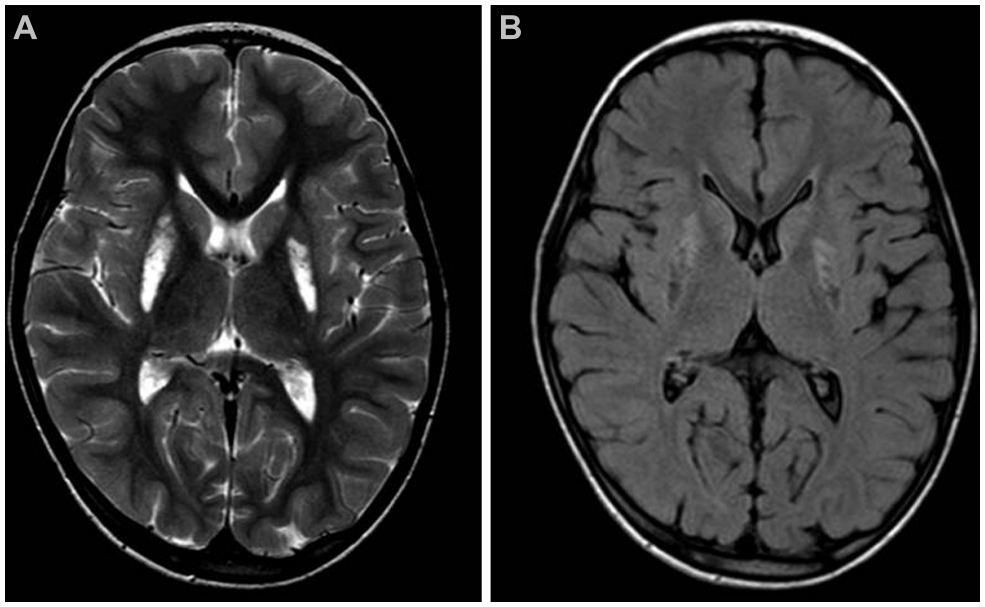
**Brain MRI.** Axial T2-weighted **(A)** and FLAIR **(B)** images of the patient, taken at 7 years of age. Note the hyperintense signals due to gliosis **(A)** and the cavitations **(B)** in the lentiform nuclei.

Brain MRI showed mild cerebellar vermis atrophy and symmetrical bilateral hyperintensity of lentiform nucleus in T2 (**Figure 1A**) and diffusion weighted images, with cavitated aspects in FLAIR sequence, as seen in necrotic brain lesions (**Figure 1B**).

Her symptoms deteriorated progressively leading to tetraparesis and severe impairment of cognitive and language performances. She is now, at 9 years of age, bed-ridden. The parents signed an informed consent, approved by the ethics committee at Makassed Hospital, in agreement with the Declaration of Helsinki.

### BIOCHEMICAL ANALYSIS

Measurement of the mitochondrial respiratory chain enzymes activity was performed by standard spectrophotometric techniques in muscle homogenate and in digitonin-treated cultures skin fibroblasts grown either in glucose-rich or in 5 mM galactose, glucose-free DMEM media for 48 h. (Bugiani et al., 2004). Oxygen consumption rate was measured using a SeaHorse FX-96 apparatus (Bioscience) in fibroblasts grown in glucose-rich DMEM medium and maximal respiration rate (MRR) was calculated as previously described (Invernizzi et al., 2012).

### MOLECULAR ANALYSIS

Total genomic DNA was extracted by standard methods from peripheral blood lymphocytes. Whole-exome sequencing (WES), using a commercial capture kit (Agilent SureSelect Human All Exon 50 Mb Kit), was performed as previously reported (Edvardson et al., 2013). Sanger sequencing of exon two of *TTC19* was performed in Pt and her parents, using the following oligonucleotides: forward (Fw) TCCTGAGCTGGAGCCTGG and reverse (Rc) AAAGCCTGGATGGATTCCAAT.

Total RNA was extracted from culture fibroblasts by RNeasy Mini Kit (QIAGEN), then 200 ng of RNA were retro-transcribed using GoScript Reverse Transcriptase (Promega), following manufacturer’s recommendations. The expression levels of *TTC19* and the housekeeping gene *GAPDH*, used for normalization, were analyzed by GoTaq qPCR Master Mix (Promega).

Primer sequences: for *TTC19*, Fw: CAAGCTGACCCCGTATAAATGC and Rc: AACAAGAAGGCCATCACTTACACTT; for *GAPDH*, Fw: CTCTGCTCCTCCTGTTCGAC and Rc: ACGACCAAATCCGTTGA.

### IMMUNOBLOT ANALYSIS

To obtain a mitochondrial enriched fraction we digitonized culture fibroblasts. Briefly, approximately 2 × 10^6^ cells were pelleted, washed twice with PBS and incubated on ice with buffer A (MOPS 20 mM, sucrose 0,25 M, pH 7.4) and digitonin 100 μg/ml for 5 min. Then sample was centrifuged at 5,000 × *g* for 3 min and pellet was incubated on ice with buffer B (MOPS tampon 20 mM, sucrose 0.25 M, EDTA Na_4_ 1 mM, pH 7.4) for 5 min, centrifuged at 10,000 × *g* for 3 min and the pellet obtained was used for western blot analysis. 50 μg of digitonin-treated culture fibroblasts from patient and controls were separated by 12% SDS-polyacrylamide gels and transferred to nitrocellulose membrane and incubated with antibodies against *TTC19* (Sigma) and SDHA (Invitrogen). Immunoblot analysis was performed with the ECL-chemiluminescence kit (Amersham).

### BLUE NATIVE GEL ELECTROPHORESIS

About 10^6^ culture fibroblasts from patient and controls were pelleted and incubated on ice with digitonin 0.4% for 10 min. Then samples were washed twice with PBS and incubated on ice with dodecylmaltoside 1% and NativePAGE Sample Buffer 1X (Invitrogen) for 15 min. After the incubation, samples were centrifuged at 20,000 × *g* for 30 min and the supernatants were measured out. Then 10 μg of supernatants plus the NativePAGE 0,25% G-250 sample additive (Invitrogen) were separated by 3–12% gradient NativePAGE Novex Bis-Tris Gels (Invitrogen) and transferred to nitrocellulose membranes and incubated with antibody against UQCRC1 (Invitrogen), NDUFS9 (Invitrogen) and SDHA (Invitrogen).

## RESULTS

### BIOCHEMICAL FINDINGS

**FIGURE 2:**
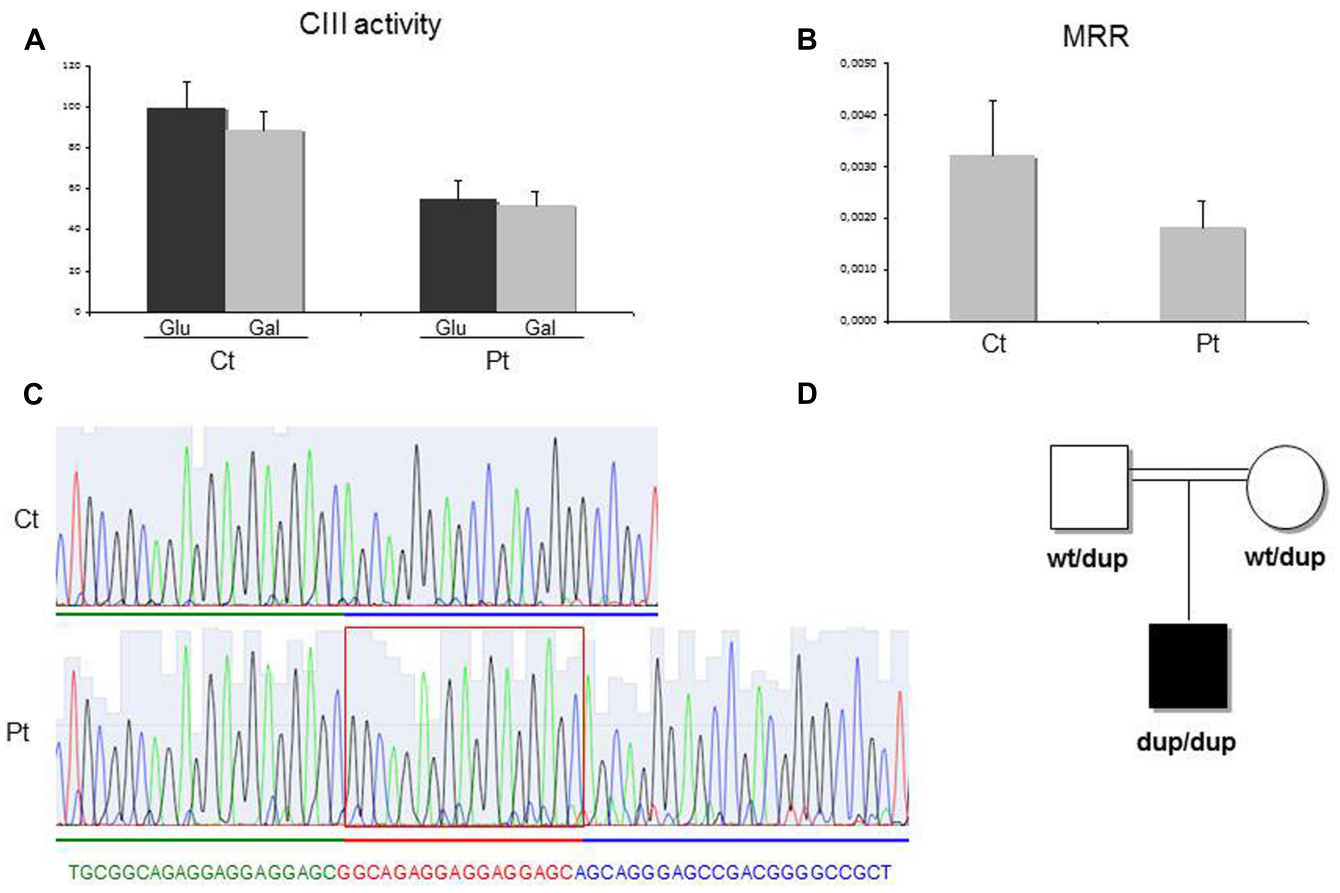
**Biochemical and genetic studies. (A)** Complex III (cIII) activity in patient (Pt) and control (Ct) fibroblasts grown in either glucose-rich or in 5 mM galactose, glucose free medium for 48 h. The specific activities were normalized for citrate synthase activity. Bars represent SD. Pt vs Ct: *p* < 0.005 (in glucose), *p* < 0.001 (in galactose). The *p*-values were obtained by unpaired, two-tail Student’s test. **(B)** Maximal respiration rate (MRR) measurements performed in Pt and Ct fibroblasts, grown in glucose medium. MRR values are expressed as pMolesO_2_/min/cells. Bars represent SD. Pt vs Ct: *p* < 0.001 (*p*-value obtained by unpaired, two tail Student’s *t*-test). **(C)** Electropherogram of the *TTC19* genomic region including the homozygous 17bp duplication (c.213_229dup) in the patient (Pt, lower panel) and in a control subject (Ct, upper panel). **(D)** Pedigree of the family. dup: allele harboring the c.213_229duplication; wt: allele harboring the wild-type *TTC19* sequence.

Spectrophotometric measurement of the activities of mitochondrial respiratory chain (MRC) complexes showed isolated, marked reduction of III in Pt muscle (28% of control mean, after normalization for the activity of citrate synthase, CS; **Table 1**). A decrease in cIII/CS was also observed in Pt fibroblasts grown in either glucose or galactose medium (55 and 52% of the control mean, respectively; **Figure 2A**). Accordingly, MRR of Pt fibroblasts was reduced compared to control cells, indicating reduced electron flow through the MRC (**Figure 2B**).

**Table 1:** Biochemical analysis of MRC complex activities in muscle homogenate.

Muscle	CS^a^	cI^b^	cII^b^	cIII^a^	cIV^c^	cV^b^
Ct value	1 ± 0.5	98 ± 46	80 ± 44	1.1 ± 0.5	9.2 ± 5.1	0.450 ± 0.229
Pt	1.3	144 (113%)	40 (84%)	**0.4 (28%)**	14 (117%)	0.631 (108%)

*Enzyme activities are expressed as: ^a^μmol/min/mg; ^b^nmol/min/mg; ^c^K/mg. In brackets the enzymatic activities normalized for citrate synthase activity (CS) and expressed as percentage of the mean control value. The values out of the control range are reported in bold*.

### GENETIC STUDIES

We performed whole exome sequencing on genomic DNA from Pt. After filtering steps to exclude common SNPs (frequency > 0.5%), we searched for homozygous variants, according to a predicted recessive mode of inheritance and because of the parents’ consanguinity. This strategy revealed the presence of a homozygous missense variant in *TTC19*. The *TTC19* open reading frame contains two putative start codons, corresponding to methionines M_1_ (NM_017775.2) and M_122_ (NM_017775.3), but the 380-aminoacids protein, starting from the M_122_, has been proposed to be the most likely human *TTC19* protein (Ghezzi et al., 2011) and now the only one reported in most of the protein databases. This is corroborated from the fact that the region upstream the M_122_ is not conserved among species. The variant found in Pt, predicted to cause the c.220G > C, p.Arg74His change in the longer isoform, was in fact localized in an untranslated region (c.–239G > C) upstream the AUG initiation codon of the standard *TTC19* isoform. To exclude a deleterious effect of this variant on the stability of *TTC19* mRNA, we quantified the levels of *TTC19* transcript in Pt fibroblasts that were comparable to control subjects. Next, we checked the coverage of all *TTC19* exons obtained by the exome analysis to identify any missing region: we noticed that exon two was poorly captured by the kit used for WES. We performed Sanger’s sequencing of exon two and identified a homozygous 17 bp duplication (c.213_229dup) in Pt (**Figure 2C**), that was present in heterozygous state in her parents (**Figure 2D**). This rearrangement is predicted to cause a frame-shift with the creation of a premature termination codon (p.Gln77Argfs^∗^30). The analysis of the *TTC19* mRNA obtained from Pt fibroblasts confirmed the presence of the 17 extra bases in the full-length transcript, without evidence of any further aberrant transcript (not shown) suggesting no influence of the duplication in the splicing processes.

### ANALYSES OF *TTC19* AND cIII ASSEMBLY

**FIGURE 3:**
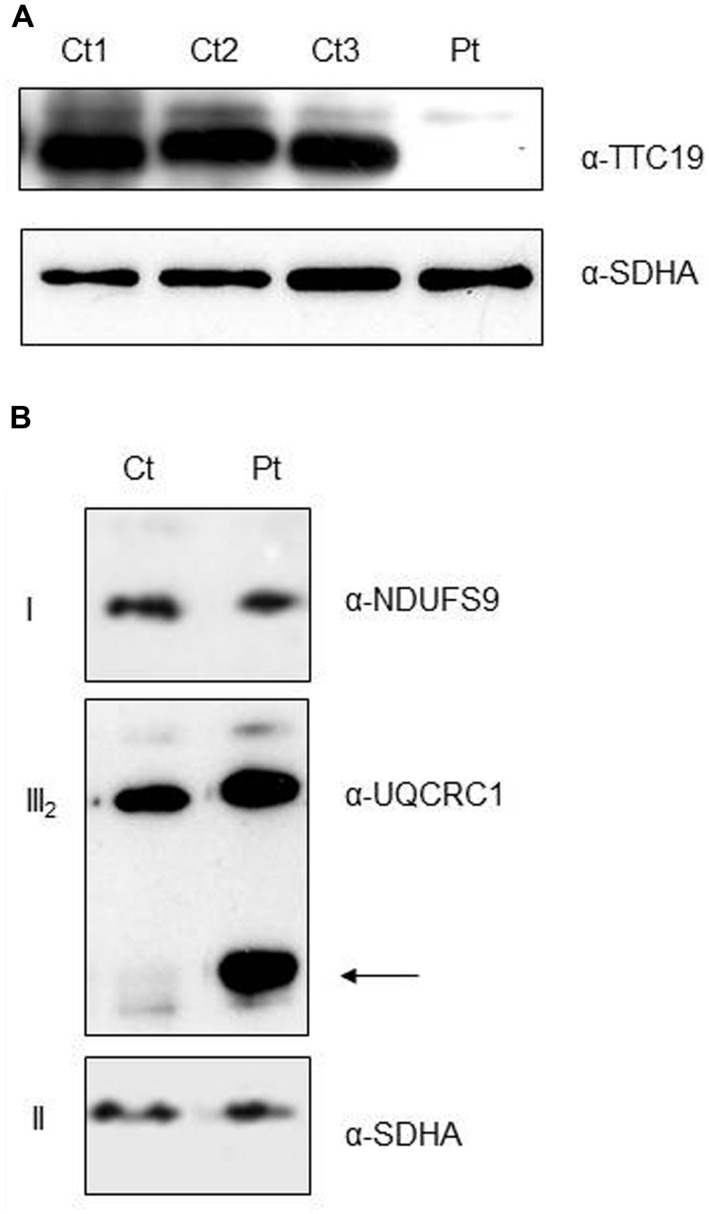
**Protein characterization and cIII assembly. (A)** Western Blot analysis of digitonin-treated fibroblasts from patient (Pt) and controls (Ct1, Ct2, Ct3) using antibodies against *TTC19* and SDHA, the latter used as loading control. **(B)** First Dimension, Blue-Native Electrophoresis analysis of fibroblasts from patient (Pt) and control (Ct). An additional band was detected (arrow) in the Pt lane using anti-complex III antibody (UQCRC1), pointing out the presence of cIII-specific assembly intermediates. Antibodies against complex I (NDUFS9) and complex II (SDHA) were used as loading controls.

As expected from the predicted consequence of the mutation on the protein, immunoblot analysis on digitonin-treated fibroblasts from Pt showed the absence of the mature *TTC19* wild-type species (**Figure 3A**). To evaluate if the absence of *TTC19* protein alters the assembly and stability of cIII we performed Blue-Native Electrophoresis analysis. No muscle was available for this assay, hence we used Pt fibroblasts. We did not observe a clear reduction in the amount of cIII holocomplex, but we detect the presence of cIII-specific assembly intermediates containing UQCRC1 subunit (**Figure 3B**), similar to those previously reported in muscle from mutant *TTC19* subjects (Ghezzi et al., 2011).

## DISCUSSION

One of the major challenges in mitochondrial medicine is to establish a correlation between disease phenotype and genotype, and then to understand the biological bases of these links. It is not rare to have multiple clinical presentations associated with mutations in the same gene. This happens also for cIII deficiency. Mutations in *MTCYB*, encoding cyt *b*, have been found linked to a wide range of neuromuscular disorders, but also to Leber hereditary optic neuropathy and to multisystem disorders (Gil Borlado et al., 2010). Mutations in *BCS1L*, encoding an assembly factor of cIII, have been associated with different clinical phenotypes such as GRACILE (acronyn for Growth Retardation, Aminoaciduria, Cholestasis, Iron overload, Lactic acidosis and Early death) syndrome, Bjornstad syndrome, neonatal proximal tubulopathy, hepatopathy and encephalopathy.

*TTC19* is considered an assembly factor for cIII since its absence has been associated with isolated cIII deficiency in *TTC19*-mutant patients and fly models (Ghezzi et al., 2011). *TTC19* has been shown to interact with cIII core subunits (UQCRC1 and UQCRC2); accordingly cIII assembly intermediates containing the same subunits were found in *TTC19*-mutant muscle. Here, we confirm the latter finding also in fibroblasts lacking *TTC19*, although in this specimen the biochemical defect was visibly less pronounced than in muscle. These results suggest that *TTC19* may play a role in an early phase of cIII assembly; alternatively, it could act as a stabilizing factor at the end of the assembly pathway and the observed cIII intermediates could be degradation products of an unstable cIII.

Mutations in *TTC19* have been associated with heterogeneous clinical presentations, including early (Ghezzi et al., 2011) or late-onset ataxia (Nogueira et al., 2013; Morino et al., 2014), cognitive impairment (Ghezzi et al., 2011), slowly progressive developmental delay/regression due to necrotizing encephalomyopathy (Leigh syndrome; Atwal, 2013) and psychiatric symptoms (Nogueira et al., 2013). MRI in these patients showed various patterns, ranging from severe olivo–ponto–cerebellar atrophy to cortical and/or cerebellar atrophy, basal ganglia and brainstem necrosis.

The patient here described presented extrapyramidal and cerebellar signs associated with cognitive impairment, symptoms described in other infantile *TTC19* patients (Ghezzi et al., 2011; Atwal, 2013), but not the clear psychiatric involvement seen in *TTC19* late-onset patients (Ghezzi et al., 2011; Nogueira et al., 2013) although behavioral and sleep abnormalities have been reported in our case. These symptoms were not rapidly and easily attributed to a specific syndrome, and also MRI patterns, characterized by cerebellar hypoplasia and bilateral basal ganglia lesions, displayed neuroradiological features seen in various mitochondrial encephalopathies, independently from the causative mutated gene.

To reach the genetic diagnosis in such cases is often very difficult. In the last years a very important support has come from new sequencing technologies such as WES. These approaches, beyond the identification of new disease genes, have allowed identifying new disease phenotypes associated with mutations in genes already known but usually associated with different clinical features.

Although the availability of these new technologies has partly overcome the need of selecting the candidate genes to be screened, for the proper interpretation of the numerous variants identified by such approach, the information on already known association between clinical phenotypes and specific mutated genes are still extremely important to reach a definite molecular diagnosis. Moreover, for strict genotype–phenotype association the classical screening of single genes remains a time and cost-effective option.

Probably new clinical presentations associated with *TTC19* mutations will be identified in the near future, thanks to WES, an unbiased approach particularly suitable for highly heterogeneous disorders with poor or still undefined genotype–phenotype correlations. Nevertheless, the biochemical cIII defect seems to be a common and constant hallmark in *TTC19* mutant patients, highlighting the crucial role of *TTC19* for proper cIII assembly or stabilization.

Hence we suggest extending the *TTC19* mutational screening, by traditional or next-generation approaches, to all patients with isolated cIII deficiency, independently from their phenotypes.

## Conflict of Interest Statement

The authors declare that the research was conducted in the absence of any commercial or financial relationships that could be construed as a potential conflict of interest.
